# Editorial: Exercise, diabetes and metabolic-associated fatty liver disease

**DOI:** 10.3389/fendo.2023.1347458

**Published:** 2023-12-19

**Authors:** Yang Sun, Chao Sun, Gang Hu, Yun Shen

**Affiliations:** ^1^ Department of Sports Medicine, Huashan Hospital, Fudan University, Shanghai, China; ^2^ Department of Gastroenterology and Hepatology, Tianjin Medical University General Hospital, Tianjin, China; ^3^ Chronic Disease Epidemiology Laboratory, Pennington Biomedical Research Center, Baton Rouge, LA, United States

**Keywords:** exercise, diabetes, fatty liver, metabolic syndrome, editorial

The interplay between lifestyle, metabolic health, and chronic diseases has become increasingly relevant in our modern health landscape (1). The prevalence of sedentary behaviors, alongside dietary excesses, has led to a surge in metabolic disorders, including diabetes and metabolic-associated steatotic liver disease (MASLD) [previously known as metabolic-associated fatty liver disease (MAFLD)] (2). These conditions, often linked and overlapping, present not only a health crisis for individuals but also a challenge for global healthcare systems. Diabetes, a major public health issue, is intricately connected to lifestyle choices. Its management and prevention are heavily influenced by physical activity and diet, necessitating a deeper understanding of these relationships. Similarly, MASLD, previously known as non-alcoholic fatty liver disease (NAFLD), has emerged as the most common liver disorder worldwide. It is closely associated with metabolic syndromes such as obesity and insulin resistance, conditions that are often precursors or companions of diabetes and future cardiovascular diseases (**Figure 1**) (3, 4).

**Figure 1:**
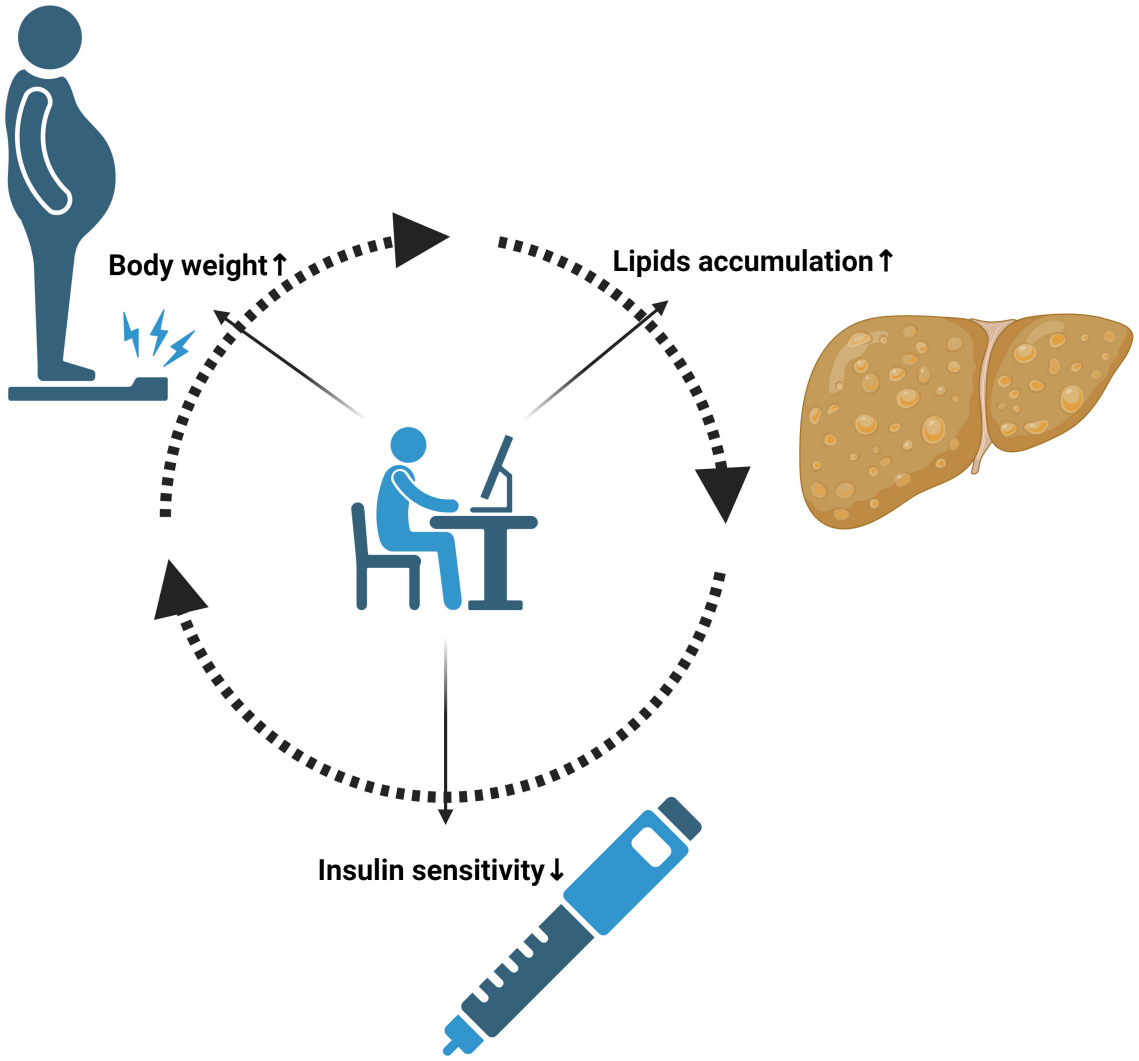
Vicious circle of Physical inactivity, Diabetes and Hepatic steatosis.

Exercise, a cornerstone of diabetes management, has also been recognized for its role in improving liver health. Physical activity, interacted with genetic susceptibility, influences glucose metabolism, insulin sensitivity, and overall metabolic function, thus playing a crucial role in managing and preventing conditions like diabetes and MASLD (5–8). However, the nuances of this relationship – the type, intensity, and duration of exercise that are most beneficial – are areas of ongoing research. Moreover, the intersection of these conditions with other comorbidities, such as cardiovascular diseases and osteoarthritis, adds layers of complexity to their management. This underscores the need for a multidisciplinary approach that considers the multifaceted nature of these diseases.

In this Research Topic, we bring together six articles that provide valuable insights into the complex relationships between exercise, diabetes, and MAFLD. Each article addresses a different aspect of this triad, contributing to a comprehensive narrative that enhances our understanding and guides future research and clinical practices.

The first article by Lei et al. explores the association between metabolic dysfunction-associated fatty liver disease and an increased risk of subclinical carotid atherosclerosis in China. This study adds to the growing body of evidence linking MAFLD with cardiovascular risks. The second article by Chai et al. reinforces the Research Topic’s overarching theme with a systematic review and meta-analysis on the effects of lifestyle interventions in adults with MAFLD. This study solidifies the role of non-pharmacological approaches in managing this condition. Shifting focus to pharmacological interventions, the third article by Kongmalai et al. presents a systematic review and network meta-analysis of randomized controlled trials on new anti-diabetic agents for treating NAFLD. This study highlights the emerging therapeutic options bridging diabetes and liver disease treatment. The fourth article introduces an innovative approach to knee osteoarthritis rehabilitation in patients with MASLD, comparing blood flow restriction training with traditional weight-bearing exercises. This multicenter randomized controlled trial offers new insights into managing MASLD-associated comorbidities. The fifth study by Vágvölgyi et al. explores the benefits of a three-month physical training program, revealing improvements in cardiovascular autonomic functions in patients with metabolic syndrome, with and without diabetes. This pilot study paves the way for future research into exercise regimes for these patients. Our last article by Bennasar-Veny et al. provides a comprehensive analysis of physical activity’s impact on glycemic control in individuals with prediabetes. This systematic review and meta-analysis emphasize the critical role of exercise in the early intervention stages of diabetes.

Together, these articles illuminate the intricate interdependencies between physical activity, metabolic health, and chronic disease management. They collectively emphasize the importance of an integrated approach to treatment and prevention, combining lifestyle interventions with medical management. This Research Topic serves as a crucial resource for clinicians, researchers, and policymakers, advocating for informed, multifaceted strategies to combat the growing challenges of diabetes and MASLD/MAFLD in the context of modern lifestyles.

## Author contributions

YaS: Writing – original draft. CS: Writing – review & editing. GH: Conceptualization, Writing – review & editing. YuS: Writing – original draft.
